# *De Novo* Production of Glycyrrhetic Acid 3-O-mono-*β*-D-glucuronide in *Saccharomyces cerevisiae*

**DOI:** 10.3389/fbioe.2021.709120

**Published:** 2021-11-23

**Authors:** Ying Huang, Dan Jiang, Guangxi Ren, Yan Yin, Yifan Sun, Tengfei Liu, Chunsheng Liu

**Affiliations:** ^1^ School of Chinese Materia Medica, Beijing University of Chinese Medicine, Beijing, China; ^2^ Key Laboratory of Biomass Chemical Engineering of Ministry of Education, College of Chemical and Biological Engineering, Zhejiang University, Hangzhou, China

**Keywords:** glycyrrhetic acid 3-O-mono-β-D-glucuronide (GAMG), *Saccharomyces cerevisiae*, cytochrome P450 enzymes (CYPs), CRISPR/Cas9, metabolic engineering

## Abstract

Glycyrrhetic acid 3-O-mono-*β*-D-glucuronide (GAMG) is a rare compound in licorice and its short supply limits the wide applications in the pharmaceutical, cosmetic, and food industries. In this study, *de novo* biosynthesis of GAMG was achieved in engineered *Saccharomyces cerevisiae* strains based on the CRISPR/Cas9 genome editing technology. The introduction of GAMG biosynthetic pathway resulted in the construction of a GAMG-producing yeast strain for the first time. Through optimizing the biosynthetic pathway, improving the folding and catalysis microenvironment for cytochrome P450 enzymes (CYPs), enhancing the supply of UDP-glucuronic acid (UDP-GlcA), preventing product degradation, and optimizing the fermentation conditions, the production of GAMG was increased from 0.02 μg/L to 92.00 μg/L in shake flasks (4,200-fold), and the conversion rate of glycyrrhetic acid (GA) to GAMG was higher than 56%. The engineered yeast strains provide an alternative approach for the production of glycosylated triterpenoids.

## Introduction

**FIGURE 1:**
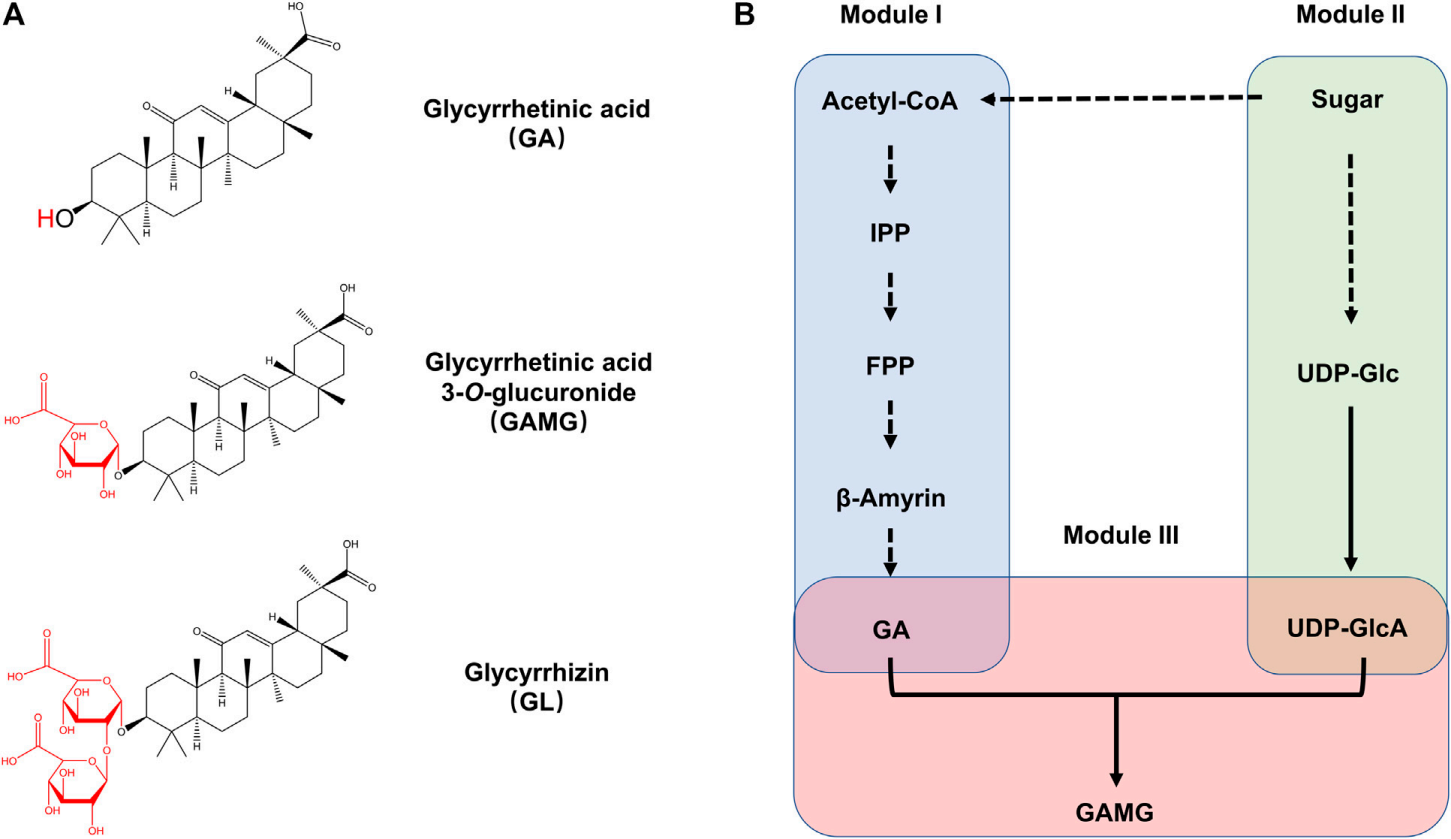
The biosynthetic pathway of GAMG. **(A)** Chemical structure of GA, GAMG, and GL. **(B)** Metabolic pathway for GAMG synthesis in *S. cerevisiae*. GAMG was synthesized from GA and UDP-GlcA by UGTs. The whole GAMG biosynthetic pathway was divided into three functional modules: the GA Module (Module I), the UDP-GlcA Module (Module II), and the glycosylation Module (Module III). IPP: isopentenyl pyrophosphate; FPP: farnesyl diphosphate; GA: glycyrrhetinic acid. GAMG: glycyrrhetic acid 3-O-mono-*β*-d-glucuronide; GL: glycyrrhizin; UDP-Glc: uridine diphosphate-glucose; UDP-GlcA: uridine diphosphate glucuronic acid.

Licorice is a popular and traditional Chinese medicinal plant and is listed in the “Medicine Food Homology” published by the Ministry of Health (China). Its roots contain plenty of strong bioactive compounds, such as triterpenoids and flavonoids (Gao et al., 2009). Two of the predominant bioactive triterpenoids are glycyrrhizin (GL) and glycyrrhetinic acid (GA), which show many valuable pharmacological properties including anti-inflammatory, anti-cancer, anti-allergic, and anti-virus effects (Wang et al., 2015), and are also widely used as natural sweeteners in candies and canned foods. In addition to the major triterpenoids, there are several minor compounds with high-value biological activities in licorice, in particular glycyrrhetinic acid monoglucuronide (3-*O*-*β*-D-glucuronopyranosyl glycyrrhetinic acid, GAMG). GAMG is composed of one molecule of GA as aglycone and one molecule of glucuronic acid (GlcA) (Figure 1A). GAMG has been shown to have similar pharmacological effects as GA and GL (Guo et al., 2018). GAMG can also be used as a natural innovative sweetener with health effects in food industry, because it is 5- and 941-times sweeter than GL and sucrose, respectively. Moreover, GAMG is a promising emulsifier or solubilization agent in various kinds of cosmetic products. Considering the great importance and the expanded market of GAMG as a valuable drug, potent food additives, and solubilization agent (Guo et al., 2018), an efficient strategy to synthesize GAMG is highly demanded for large-scale production of GAMG in industry.

GAMG are mainly obtained through plant extraction, chemical synthesis, and biotransformation. The extremely low GAMG content in licorice reduces the efficiency of GAMG extraction (<0.1 mg/g) (Song et al., 2017), and wild licorice overharvesting results in environmental damage and even desertification. Chemical synthesis of GAMG suffers from complicated process, high cost, and environmental pollution (Guo et al., 2018). Recently, GAMG and lignocellulosic enzyme were produced by the licorice straw as a medium for *via* solid-state fermentation (SSF) of endophytic fungus *Chaetomium globosum* DX-THS3(Gao et al., 2021), however the 90% GL transformed to GAMG needs 15 days. Currently, biotransformation with GL as the substrate is the major approach for GAMG production, unfortunately not fully addressing the source and environmental damage concerns. In other words, novel strategies for GAMG production should be developed to satisfy the growing market. Recently, heterologous biosynthesis of various natural products with plant origins, such as terpenoids (artemisinic acid, GA, and crocetin) (Paddon et al., 2013; Zhu et al., 2017; Liu et al., 2020), flavonoids (liquiritin, anthocyanin, and scutellarein) (Wang et al., 2016; Levisson et al., 2018; Yin et al., 2020), and alkaloids (vindoline and strictosidine) (Brown et al., 2015; Qu et al., 2015), have been reported in engineered yeast strains. In particular, GA, the precursor of GAMG, has been successfully biosynthesized in *Saccharomyces cerevisiae* by introducing the biosynthetic pathway including the *β-*amyrin synthase, two cytochrome P450 enzymes (CYPs, *CYP88D6* and *CYP72A154*) (Seki et al., 2008; Seki et al., 2011), with 2, 3-oxidosqualene as the biosynthesis precursor. These studies indicated the possibility of *de novo* biosynthesis of GAMG if a GA-specific glycosyltransferase is available.

GuUGT73F15 has been firstly shown to transfer a GlcA moiety to the 3-position of GA to produce GAMG *in vitro*. However, its application in *de novo* biosynthesis of GAMG in yeast is yet to be explored and the construction of a GAMG-producing yeast strain by genome editing has not been reported. Herein, the present study was aimed to construct a GAMG-producing strain using the CRISPR/Cas9 technology in *S. cerevisiae*. First, GuUGT73F15 was confirmed to catalyze the glycosylation of GA to generate GAMG in yeast. After the proof-of-concept verification, the GA biosynthetic pathway and the UDP-GlcA biosynthetic pathway were introduced and optimized to achieve *de novo* production of GAMG. Followed by the optimization of fermentation conditions, the production of GAMG and the glycosylation of GA to GAMG was further improved. Our study lays a solid foundation for the production of other glycosylated-GA derivatives in the future.

## Materials and Methods

### Plasmid Construction

*Escherichia coli* strain TransT1 (TransGen, Beijing, China) was used for plasmid maintenance and propagation. Genes involved in the biosynthesis of GAMG (Supplementary Table S1) were cloned into the multiple cloning sites (*BamhI*/*XhoI*; *NotI*) of the pESC series plasmids or pUMI-22, using a seamless cloning kit (TransGen Biotech). The Benchling CRISPR tool (https://benchling.com/crispr/) was used to design gRNAs. PCR products were obtained by gene amplification (KOD Neo Plus; TOYBO, Tokyo, Japan) and gel purification (GENE GEL JET; Thermofisher Scientific, Shanghai, China). All assembled plasmids and the gRNA plasmids were sequenced by TSINGKE Biological Technology (Hangzhou, China). All the plasmids and primers used in this study were listed in Supplementary Tables S2, S3 respectively. Oligos used to construct gRNAs were listed in Supplementary Table S4.

### Strain Construction

The CRISPR/Cas9-mediate genome editing technology was used for the construction of engineered yeast strains (Lian et al., 2018). Yeast strains were transformed using the classical LiAC/ssDNA/PEG protocol, and the transformants were selected on the appropriate agar plates. DNA donors were amplified from the constructed plasmids with 40 bp-homology arms to the yeast genomic DNA integration sites. All yeast strains used in this study were summarized in Supplementary Table S5.

### Growth and Culture Conditions

*E. coli* recombinant strains were cultured in Luria-broth medium (OXIOD Biotech, London, United Kingdom) with 100 μg/ml ampicillin at 37 C. Yeast strains were routinely cultivated in yeast peptone dextrose (YPD) medium. Recombinant yeast strains were selected on synthetic complete medium (SED) consisting of 0.17% YNB (BD Diagnostics), 0.1% mono-sodium glutamate, and 0.06% appropriate amino acid drop out mix (MP Biomedicals, Solon, OH, United States), supplemented with 2% dextrose and 200 μg/ml G418 (Sangon Biotech, Shanghai, China). Positive yeast transformants were precultured in YPD for 24 h and then inoculated into 20 ml YPD medium in a 250 ml shake flask. After growing at 30°C and 250 rpm for 24 h, the yeast strains were transferred to a new 250 ml shake flask containing YP medium with the appropriate carbon source (glucose, galactose, or glycerol). GAMG shake flask fermentation was performed at 30°C and 250 rpm for 6 days.

### Sample Preparation

For product quantification, yeast cells were harvested from 20 ml culture by centrifugation at 6,000 rpm, washed twice, and vacuum freeze-dried at to a constant weight. Then dry cell pellets were transferred into a 2 ml tube with 0.8 ml of an acetone: methanol mixture (1:1), and the resuspended yeast cells were crushed three times with glass beads. The samples were centrifuged at 12,000 rpm for 1 min, and the supernatants filtered with a 0.22 μm syringe filter were analyzed by LC-MS. The standard of chemicals were purchased from Yuanye Bio-tech Co., Ltd (Shanghai, China) and Chengdu DeSiTe Biological Technology Co., Ltd (Chengdu, China).

### Analytical Methods

GAMG and GA were analyzed by LC-ESI-MS (SHIMADZU, Tokyo, Japan). 2 μL filtrate was injected and separated using a HyPURITY™ C18 HPLC (150 mm × 4.6 mm, 3 μm, Thermofisher Scientific) column with an ESI ion source equipped with a triple quadrupole mass analyzer. The mobile phase comprised of 0.1% (*v/v*) formic acid solution (A) and acetonitrile (B) at a flow rate of 0.2 ml/min. The gradient elution program was as follows: 0–1 min, 2% B; 1–10 min, 2.0%–80% B; 10–15 min, 80%–90% B; 15–18 min, 90%–98% B; 18–35 min, 98%–2% B; 35–40 min, 2% B. The column temperature was set at 30°C. GAMG and GA were monitored using multiple-reaction monitoring (MRM) in the negative ion mode with the following parameters: GAMG with a collision energy of −22 eV and an m/z transition from 647.35 to 453.25; GA with a collision energy of −40 eV and an m/z transition from 471.35 to 351.05.

## Results

The biosynthesis of GAMG in yeast mainly consisted of pathways from acetyl-CoA to GA and from sugar to UDP-glucuronic acid, followed by *O*-glycosylation by the glycosyltransferase (Figure 1B). In this case, the GAMG biosynthetic pathways were divided into three functional modules, the GA module (Module I), the UDP-GlcA module (Module II), and the glycosylation module (Module III). In the present study, the three biosynthetic modules were tested and optimized individually and then combined to achieve *de novo* biosynthesis of GAMG.

### Functional Verification of the Module III in Yeast

To verify the feasibility of the designed pathway, the glycosylation module (Module III) was firstly tested. The synthetic codon optimized Gu*UGT73F15* and At*UDH* (encoding UDP-glucose dehydrogenase from *Arabidopsis thaliana* to catalyze the generation of UDP-GlcA from UDP-Glc) were consecutively cloned into the pESC-LEU plasmid. The engineered strains CI04-1-1, harboring Gu*UGT73F15* and At*UDH* expression cassettes, was cultivated in YP-Galactose medium supplemented with GA, and the supernatant as well as cell pellets were analyzed by LC-MS. A new compound (peak 1), with the same retention time and MS spectrum as those of the GAMG standard, was detected in the samples (cell pellets) only when GA was supplemented (Supplementary Figure S1). Moreover, another new compound (peak 3) [*m/z* 631.6] was also identified as GA-monoglucoside by MS spectra. These were consistent with the result of Chen et al. (2019) that GuUGT73F15 was able to accept UDP-GlcA and UDP-Glc as the sugar donor for the glycosylation of GA. Although GuUGT73F15 was able to accept UDP-Glc as one of the sugar donor, it also could produce a proportion of GAMG when UDP-Glc appeared *in vivo*. Therefore, Gu*UGT73F15* and At*UDH* were used in subsequent studies.

### Improving GAMG Production *via* Biosynthetic Pathway Optimization

**FIGURE 2:**
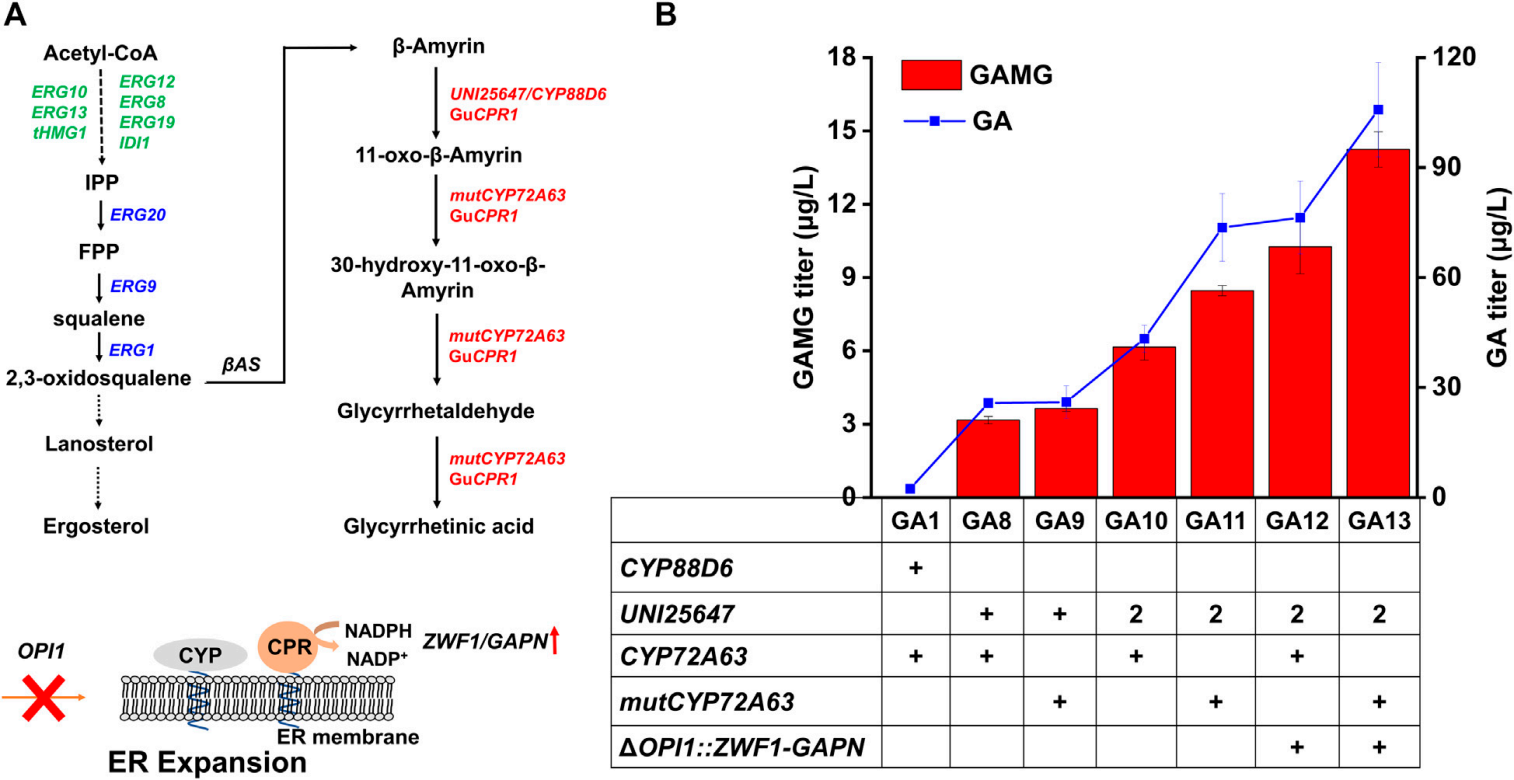
Optimization of the GA module (module I) to improve GAMG production. **(A)** Optimization of GA biosynthetic pathway for improved GAMG production. Endogenous genes overexpressed in the parent strain BY4741-C-04 were shown in green, endogenous genes overexpressed in the present study were shown in blue, and heterologous genes overexpressed to synthesize GA were shown in red. *ZWF1* and *GAPN* were overexpressed to enhance NADPH supply and *OPI1* was disrupted to provide more space for the folding of CYPs *via* ER membrane expansion. **(B)** Comparison of GA and GAMG production in engineered *S. cerevisiae* strains. ERG10: acetyl-CoA acetyltransferase; ERG13: hydroxymethylglytaryl-CoA synthase; tHMG1: truncated-3-methylglutaryl-CoA reductase; ERG12: mevalonate kinase; ERG8: phosphomevalonate knase; ERG19: diphosphomevalonate decarboxylase; IDI1: dimethylallyl diphosphate isomerase; ERG20: farnesyl pyrophosphate synthase; ERG9: squalene synthase; ERG1: squalene epoxidase; βAS: β-amyrin synthase; GuCPR1: cytochrome P450 reductase from *Glycyrrhiza uralensis*; UNI25647: β-amyrin 11-oxidase; mutCYP72A63: the T388S/W205R mutant of 11-oxo-β-aymrin 30-oxidase; GAPN: NADP^+^-dependent glyceraldehyde-3-phosphate dehydrogenase from *Streptococcus mutans*; OPI1: OverProducer of Inositol; ZWF1: glucose-6-phosphate dehydrogenase; ER: endoplasmic reticulum. Error bars represented SD of biological triplicates.

After the proof-of-concept verification, the previously constructed strain BY4741-C-04, where each gene of the mevalonate (MVA) pathway was overexpressed to enhance the accumulation of isopentenyl pyrophosphate (IPP), was used as the parent strain for GAMG production (Wang C. et al., 2019) (Supplementary Table S5). Further introduction of GAMG biosynthetic pathway genes (Figure 2A), including *ERG1*, the *β-*amyrin synthase encoding gene (*βAS*), two cytochrome P450 enzyme (CYP) encoding genes (*CYP88D6* and *CYP72A63*), the CYP reductase encoding gene (Gu*CPR1/*Cr*CYB5*), as well as *ERG20* and *ERG9*, into the chromosome of CI04-1-1 (GA1, Supplementary Table S5), GA and GAMG was accumulated to 2.4 μg/L and 0.02 μg/L, respectively. Although *de novo* biosynthesis of GAMG was achieved, the production level was extremely low, indicating that the GA module (Module I) and the UDP-GlcA module (Module II) should be further optimized to redirect the metabolic fluxes towards GAMG biosynthesis.

Subsequently, the GA module (Module I) was optimized to enhance the production of GA and GAMG. Previous studies found the two CYPs (GuCYP88D6 and MtCYP72A63) to be rate-limiting for GA biosynthesis. Compared with the two CYPs used for the construction of GA1, CYPs with higher catalytic efficiency have been reported, such as UNI25647 with the C-11 hydroxylation activity (Zhu et al., 2017) and CYP72A63 mutant (T388S/W205A) with C-30 hydroxylation activity (Brown et al., 2015; Sun et al., 2020). Therefore, *CYP88D6* and *CYP72A63* in GA1 was replaced by *UNI25647* and mut*CYP72A63* to improve the production of GAMG in yeast. The replacement of *CYP88D6* with *UNI25647* resulted in the construction of strain GA8, whose production of GA and GAMG was increased approximately 9.8- and 154- fold to 25.76 μg/L and 3.17 μg/L, respectively. GA10 was constructed by integrating another copy of *UNI25647* expression cassette into GA8, and the production of GA and GAMG were further improved by 1.68- and 1.94-fold, respectively. The replacement of *CYP72A63* in strain GA8 and GA10 by its mutant (T388S/W205A) resulted in the construction of strain GA9 and GA11, whose production of GA and GAMG were even higher (Sun et al., 2020). Compared with the parent strain GA1, the production of GA and GAMG in GA11 were increased by 31- and 419-fold, respectively, *via* molecular engineering of the two CYP encoding genes (Zhu et al., 2017; Sun et al., 2020).

### Engineering CYPs Microenvironment and Increasing NADPH Supply for Increased GAMG Production

Besides molecular engineering of CYPs, the microenvironment for CYP folding and catalysis could be engineered for optimal performance as well. Considering that most plant CYPs were endoplasmic reticulum (ER) membrane localized and required NADPH for electron transfer (Hausjell et al., 2018), ER expansion and enhanced NADPH supply were commonly employed to improve the enzymatic activities of CYPs. Previous studies demonstrated that the deletion of *OPI1* enabled ER expansion and then provided more space for the folding of CYPs (Arendt et al., 2017; Kim et al., 2019). Additionally, NADPH supply could be increased by introducing *GAPN* (NADP^+^-dependent glyceraldehyde-3-phosphate dehydrogenase from *Streptococcus mutans*) (Chen et al., 2014) and overexpressing *ZWF1* (Kwak et al., 2019). Thus, strain GA13 was constructed by inserting the *GAPN* and *ZWF1* expression cassettes into the *OPI1* locus of GA11. The production of GA and GAMG in GA13 was 1.4- and 1.7-fold higher than those of GA11 (Figure 2B, 105.78 μg/L and 14.24 μg/L), indicating that engineering of the CYP microenvironment was beneficial for improving the production of GA and GAMG in yeast. Although the production of GA and GAMG were dramatically increased, the conversion rate (conversion rate was calculated by the molar ratio of GA to GAMG) of GA to GA was only ∼13%, indicating that the glycosylation of GA to GAMG has potential to be further improved.

### Increasing UDP-GlcA Supply for Increased GAMG Production

**FIGURE 3:**
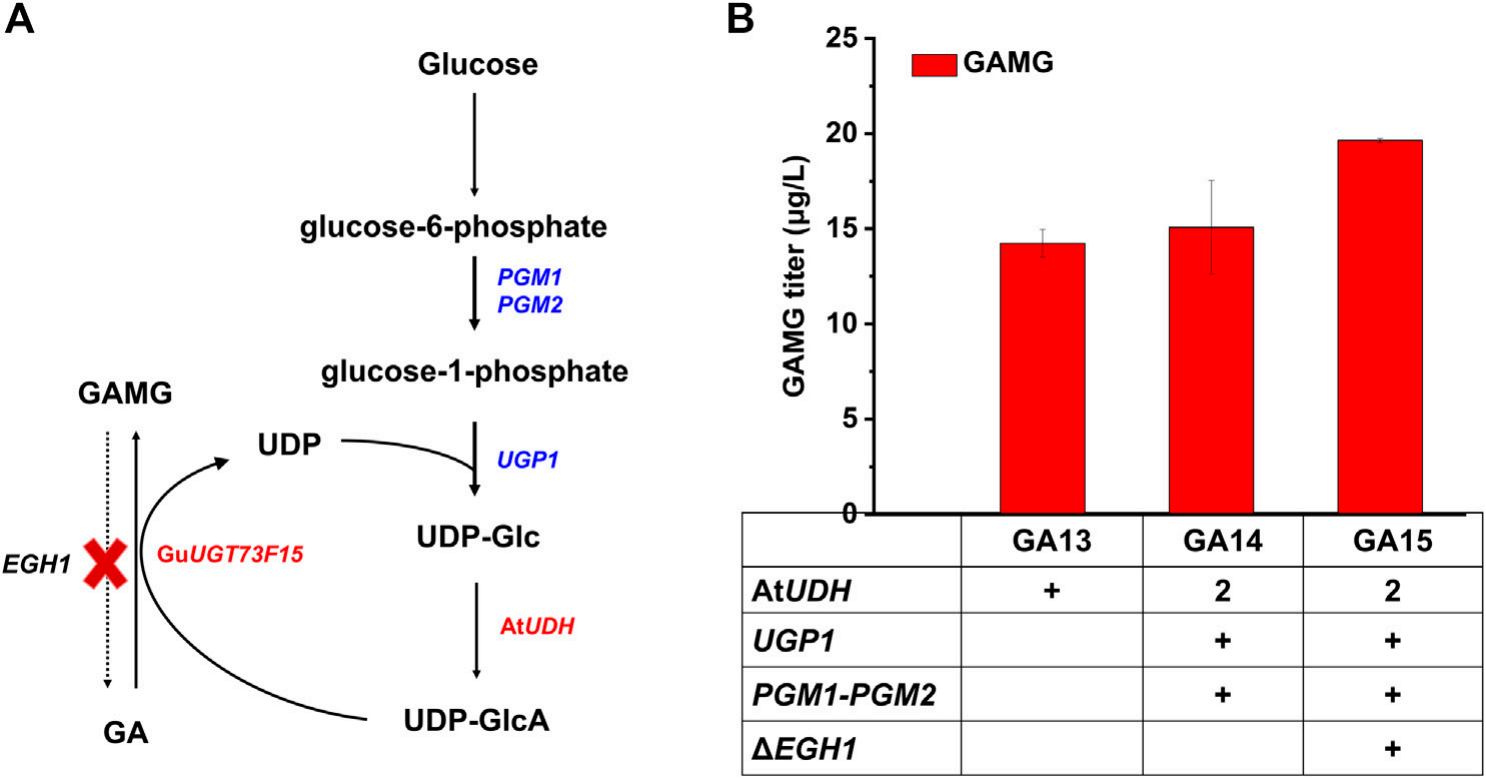
Optimization of the UDP-GlcA module (module II) for improving GAMG production, including the prevention of GAMG degradation and enhancement of UDP-GlcA supply. **(A)** UDP-GlcA biosynthetic pathway and its involvement in GA glycosylation. *PGM1*, *PGM2*, *UGP1,* and At*UDH* were overexpressed to enhance UDP-GlcA supply. *EGH1* was deleted to prevent GAMG degradation. **(B)** Comparison of GA and GAMG production in engineered *S. cerevisiae* strains. PGM1: phosphoglucomutase 1; PGM2: phosphoglucomutase 2; UGP1: UDP-glucose pyrophosphorylase; AtUDH: UDP-glucose dehydrogenase from *A. thaliana*; GuUGT73F15: UDP-glucuronyltransferase. Error bars represented SD of biological triplicates.

Then, the UDP-GlcA module (Module II) was explored for the optimization of the GAMG biosynthetic pathway, including the enhanced supply of UDP-GlcA and inhibition of GAMG hydrolysis (Figure 3A). The supply of UDP-Glc could be enhanced by overexpressing the endogenous *PGM1*, *PGM2,* and *UGP1* (Zhuang et al., 2017; Wang et al., 2019b; Wang et al., 2020). Therefore, these three genes together with At*UDH* were integrated into strain GA13, resulting in the construction of strain GA14. Unfortunately, the production of GAMG was not significantly increased (Figure 3B), we speculate that overexpressing the endogenous *PGM1*, *PGM2,* and *UGP1* is more benefit for the glucose promoter direct genes than the genes are driven by galactose promoters. In addition, EGH1 (ergosteryl-β-glucosidase) was reported to hydrolyze ergosteryl-β-glucoside and those with similar structures (Watanabe et al., 2015). For example, the deletion of *EGH1* prevented the degradation Rh2, and accordingly enhanced Rh2 accumulation in yeast (Zhuang et al., 2017). Considering the similar structure of GAMG and Rh2, the deletion of *EGH1* was expected to prevent the degradation of GAMG. Hence, GA14 was further engineered by the deletion of *EGH1* (GA15). The production of GAMG in GA15 was increased to 19.63 μg/L (Figure 3B). Overall, the conversion rate of GA to GAMG was improved to 20.22% *via* the enhancement of UDP-GlcA supply and prevention of GAMG degradation.

### Various Ratios of Glycerol, Glucose and Galactose Studies on GAMG Production

**FIGURE 4:**
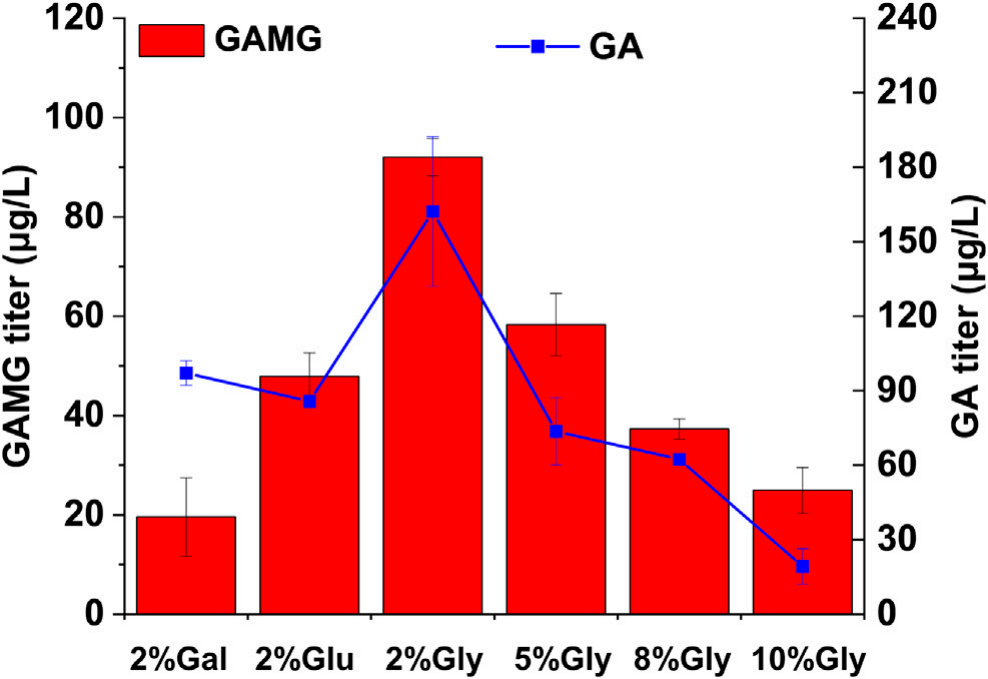
Optimization of carbon sources for enhanced GAMG production in yeast strain GA15. GAMG and GA fermentation were carried out in YP medium supplemented with 2% galactose, 2% glucose, or different concentration of glycerol. Error bars represent SD of biological triplicates. Gal: galactose; Glu: glucose; Gly: glycerol.

Finally, the effect of carbon sources on GAMG production was investigated using the highest GAMG-producing strain GA15(Supplementary Figure S2). Considering the disruption of *GAL80* in all the engineered strains, heterologous genes under the control of galactose-inducible promoters could be induced even without the presence of galactose. Thus, glucose, a much cheaper carbon source, was evaluated for GAMG fermentation. Interestingly, the replacement of carbon source in the fermentation medium from galactose to glucose resulted in more than 2-fold improvement in the production of GAMG (Figure 4), probably due to better supply of UDP-GlcA and accordingly enhanced GA glycosylation. In addition, a previous study demonstrated that the supplementation of glycerol into the fermentation medium increased noscapine production substantially, probably due to the generation of the essential cofactor NADPH and enhanced stability of the subcellular membrane localized proteins (Gekko and Serge, 1981; Crowe et al., 1984; Crowe et al., 1988; Sato et al., 1996; Costenoble et al., 2000; Bolen, 2001; Diamant et al., 2001; Meng et al., 2001; Perlmutter and David, 2002; Li et al., 2018). Therefore, glycerol at different concentrations (0, 2, 5, 8, and 10%) was supplemented to investigate GAMG production. As shown in Figure 4, 2% glycerol supplementation led to the highest production of GAMG (92.00 μg/L). Moreover, the conversion yield of GA to GAMG was improved to about 56%. However, higher glycerol concentration resulted in impaired GA and GAMG production, which was consistent with the previous study that glycerol concentration should be maintained at an appropriate level for optimal production of noscapine (Li et al., 2018).

## Discussion

Although systematic metabolic engineering strategies have been employed in the present study, the production of GA and GAMG was still much lower than other triterpenoids (include GA) reported previously (Zhu et al., 2017; Zhuang et al., 2017; Wang C. et al., 2019; Zhang et al., 2020). The discrepancy in GA production might result from the use of yeast strains with different genetic backgrounds. It is noteworthy that the highest GA producing strain was achieved in a diploid yeast strain. Zhu et al. compared the production of GA in a diploid strain (i.e. INVSc1) and a haploid strain (CEN.PK2-1C) and found that the diploid yeast strain was much more appropriate for GA production (Zhu et al., 2017). In the present study, an IPP accumulating strain, derived from the haploid strain BY4742, was used as the parent strain, with a focus on the engineering of microenvironment for CYPs and UDP-GlcA pathway to enhance the production of GAMG (Yang et al., 2021). Therefore, the transfer of the metabolic engineering strategies to a diploid yeast strain is expected to further improve GAMG production and will be explored in the following studies. Meanwhile we notice that the other three glycosyltransferases (*GuCSyGT*, *GmCSyGT1* and *LjCSyGT1*) recently have been reported to specifically transfer GlcA to GA to produce GAMG by using plasmid system (Chen et al., 2019; Chung et al., 2020; Jozwiak et al., 2020) except for *GuUGT75F15*, and these results implied the *GuCSyGT* has better performance on *de novo* production GAMG in yeast. And in the future study, it is very essential to apply *GuCSyGT* for improving the production of GAMG in the yeast cell factories, and fed-batch fermentation in the bioreactor will be carried out to improve GAMG production.

In conclusion, *de novo* biosynthesis of GAMG in yeast was reported for the first time aided by the CRISPR/Cas9 genome editing technology. Through a combination of biosynthetic pathway optimization, enhancement of NADPH supply, expansion of ER membranes, prevention of GAMG degradation, and fermentation optimization, the production of GAMG was increased for more than 4,600-fold to a final titer of 92 μg/L in shake flasks. The present study provides a solid foundation for the production of other glycosylated triterpenoids in yeast.

## Data Availability

The datasets presented in this study can be found in online repositories. The names of the repository/repositories and accession number(s) can be found in the article/Supplementary Material.
