# Supplementary material for: De Novo Production of Glycyrrhetic Acid 3-O-mono-β-D-glucuronide in Saccharomyces cerevisiae
Source: Front Bioeng Biotechnol. 2021 Nov 23;9:709120. doi: 10.3389/fbioe.2021.709120 (PMC8650490; doi:10.3389/fbioe.2021.709120)
Supplement: Supplementary file 1 [file DataSheet1.doc]

Supplementary Material

***De novo* Production of glycyrrhetic acid 3-O-mono-*β*-D-glucuronide in *Saccharomyces cerevisiae***

Ying Huang^1^, Dan Jiang ^1^, Guangxi Ren^1^, Yan Yin^1^, Yifan Sun^1^, Tengfei Liu^2*^, Chunsheng Liu^1*^

^1^ School of Chinese Materia Medica, Beijing University of Chinese Medicine, Beijing 102488, China

^2^ Key Laboratory of Biomass Chemical Engineering of Ministry of Education, College of Chemical and Biological Engineering, Zhejiang University, Hangzhou 310027, China

^*^ Corresponding authors:

Pro. Chunsheng Liu

Email: [max_liucs@263.net](mailto:max_liucs@263.net)

Dr. Tengfei Liu

Email: ltfe@zju.edu.cn

**Supplementary Tables**

**Supplementary Table S1** DNA sequences of genes used in this study

| Genes | DNA sequences |
| --- | --- |
| *CYP88D6(Huang et al., 2019)* | ATGGAAGTACATTGGGTTTGCATGTCCGCTGCCACTTTGTTGGTATGCTACATTTTTGGAAGCAAGTTTGTGAGGAATTTGAATGGGTGGTATTATGATGTAAAACTAAGAAGGAAAGAACACCCACTACCCCCAGGTGACATGGGATGGCCTCTTATCGGCGATCTATTGTCCTTCATCAAAGATTTCTCATCGGGTCACCCTGATTCATTCATCAACAACCTTGTTCTCAAATATGGACGAAGTGGTATCTACAAGACTCACTTGTTTGGGAATCCAAGCATCATTGTTTGTGAGCCTCAGATGTGTAGGCGAGTTCTCACTGATGATGTGAACTTTAAGCTTGGTTATCCAAAATCTATCAAAGAGTTGGCACGATGTAGACCCATGATTGATGTCTCTAATGCGGAACATAGGCTTTTTCGACGCCTCATTACTTCCCCAATCGTGGGTCACAAGGCGCTAGCAATGTACCTAGAGCGTCTTGAGGAAATTGTGATCAATTCGTTGGAAGAATTGTCCAGCATGAAGCACCCCGTTGAGCTCTTGAAAGAGATGAAGAAGGTTTCCTTTAAAGCCATTGTCCACGTCTTCATGGGCTCTTCCAATCAGGACATCATTAAAAAAATTGGAAGTTCGTTTACTGATTTGTACAATGGCATGTTCTCTATCCCCATTAACGTACCTGGTTTTACATTCCACAAAGCACTCGAGGCACGTAAGAAGCTAGCCAAAATAGTTCAACCCGTTGTGGATGAAAGGCGGTTGATGATAGAAAATGGTCCACAAGAAGGGAGCCAAAGAAAAGATCTTATTGATATTCTTTTGGAAGTCAAAGATGAGAATGGACGAAAATTGGAGGACGAGGATATTAGCGATTTATTAATAGGGCTTTTGTTCGCTGGCCATGAAAGTACAGCAACCAGTTTAATGTGGTCAATTACGTATCTTACACAGCATCCCCATATCTTGAAAAAGGCTAAGGAAGAGCAGGAAGAAATAACGAGGACAAGATTTTCCTCGCAGAAACAATTAAGTCTTAAGGAAATTAAGCAAATGGTTTATCTTTCTCAGGTAATTGATGAAACTTTACGATGTGCCAATATTGCCTTTGCAACTTTTCGAGAGGCAACTGCTGATGTGAACATCAATGGTTATATCATACCAAAGGGATGGAGAGTGCTAATTTGGGCAAGAGCCATTCATATGGATTCTGAATATTACCCAAATCCAGAAGAATTTAATCCATCGAGATGGGATGATTACAATGCCAAAGCAGGAACCTTCCTTCCTTTTGGAGCAGGAAGTAGACTTTGTCCTGGAGCCGACTTGGCGAAACTTGAAATTTCCATATTTCTTCATTATTTCCTCCGTAATTACAGGTTGGAGAGAATAAATCCAGAATGTCACGTTACCAGCTTACCAGTATCTAAACCCACAGACAATTGTCTCGCTAAGGTGATGAAGGTCTCATGTGCTTAG |
| *UNI25647(Zhu et al., 2017)* | ATGGAAGTACATTGGGTTTGCATGTGCGCTGCCACTTTGTTGGTATGCTACATTTTTGGAAGCAAGTTTGTGAGGAATTTGAATGGGTGGTATTATGATGTAAAACTAAGAAGGAAAGAACACCCACTACCCCCAGGTGACATGGGATGGCCTCTTATGGGCAATCTATTGTCCTTCATCAAAGATTTCTCATCGGGTCACCCTGATTCATTCATCAACAACCTTGTTCTCAAATATGGACGAAGTGGTATCTACAAGACTCACTTGTTTGGGAATCCAAGCATCATTGTTTGCGAGCCTCAGATGTGTAGGCGAGTTCTCACTGATGATGTGAACTTTAAGCTTGGTTATCCAAAATCTATCAAAGAGTTGGCACGATGTAGACCCATGATTGATGTCTCTAATGCGGAACATAGGCTTTTTCGACGCCTCATTACTTCCCCAATCGTGGGTCACAAGGCGCTAGCAATGTACCTAGAACGTCTTGAGGAAATTGTGATCAATTCGTTGGAAGAATTGTCCAGCATGAAGCACCCCGTTGAGCTCTTGAAAGAGATGAAGAAGGTTTCCTTTAAAGCCATTGTCCACGTTTTCATGGGCTCTTCCAATCAGGACATCATTAAAAAAATTGGAAGTTCGTTTACTGATTTGTACAATGGCATGTTCTCTATCCCCATTAACGTACCTGGTTTTACATTCCACAAAGCACTCGAGGCACGTAAGAAGCTAGCCAAAATAGTTCAACCCGTTGTGGATGAAAGGCGGTTGATGATAGAAAATGGTCAACAAGAAGGGGACCAAAGAAAAGATCTTATTGATATTCTTTTGGAAGTCAAAGATGAGAATGGACGAAAATTGGAGGACGAGGATATTAGCGATTTATTAATAGGGCTTTTGTTTGCTGGCCATGAAAGTACAGCAACCAGTTTAATGTGGTCAATTACATATCTTACACAGCATCCCCATATCTTGAAAAAGGCTAAGGAAGAGCAGGAAGAAATAATGAGGACAAGATTGTCCTCGCAGAAACAATTAAGTTTTAAGGAAATTAAACAAATGGTTTATCTTTCTCAGGTAATTGATGAAACTTTACGATGTGCCAATATTGCCTTTGCAACTTTTCGAGAGGCAACTGCTGATGTGAACATCAATGGTTATATCATACCAAAGGGATGGAGAGTGCTAATTTGGGCAAGAGCCATTCATATGGATTCTGAATATTACCCAAATCCAGAAGAATTTAATCCATCGAGATGGGATGATTACAATGCCAAAGCAGGAACCTTCCTTCCTTTTGGAGCAGGAAGTAGACTTTGTCCTGGAGCCGACTTGGCGAAACTTGAAATTTCCATATTTCTTCATTATTTCCTCCTTAATTACAGGTTGGAGCGAGTAAATCCAGAATGTCATGTTACCAGCTTACCAGTATCTAAGCCCACAGACAATTGCCTCGCTAAGGTGATGAAGGTCTCATGTGCTTAG |
| *CYP72A63(Zhu et al., 2017)* | ATGGAAGTTTTCATGTTCCCAACTGGTACTACTGTTATCATCTCTGTTTTGTCTGTTTTGTTGGCTGTTATCCCATGGTACTTGTTGAACAAGTTGTGGTTGAAGCCAAAGAGATTCGAAAAGTTGTTGAAGGCTCAAGGTTTCCAAGGTGAACCATACAACTTGTCTGTTTTGAAGGACAAGTCTAAGCAAAACTACATGTTGAAGTTGCAACAAGAAGACAAGTCTAAGTCTATCGGTTTGTCTAAGGAAGCTGCTCCATCTATCTTCACTCCAGTTCACCAAACTGTTAGAAAGTACGGTAACAACTCTTTCTTGTGGGAAGGTACTACTCCAAGAGTTATCATCACTGACCCAGACCAAATCAAGGACGTTTTCAACAAGATCGACGACTTCCCAAAGCCAAAGTTGAGATCTATCGCTAAGTACTTGTCTGTTGGTATCTTGGACCACGAAGGTAAGAAGTGGGCTAAGCACAGAAAGATCGCTAACCCAGCTTTCCACTTGGAAAAGTTGAAGGTTATGTTGCCAGCTTTCTCTCACTCTTGTAACGAAATGATCTCTAAGTGGAAGGAATTGTTGTCTTCTGACGGTACTTGTGAAATCGACGTTTGGCCATCTTTGCAAAACTTCACTTGTGACGTTATCTCTAGAACTGCTTTCGGTTCTTCTTACGCTGAAGGTACTAAGTTGTTCCAATTGTTGAAGAAGCAAGGTTTCTTGTTGATGACTGGTAGACACACTAACAACCCATTGTGGGGTTTGTTGGCTACTACTACTAAGACTAAGATGAAGGAAATCGACAGAGAAATCCACGACTCTTTGGAAGGTATCATCGAAAAGAGAGAAAAGGCTTTGAAGAACGGTGAAACTACTAACGACGACTTGTTGGGTATCTTGTTGCAATCTAACCACGCTGAAAAGCAAGGTCAAGGTAACTCTAAGAACATCGGTATGACTACTCAAGACGTTATCGACGAATGTAAGTTGTTCTACTTGGCTGGTCAAGAAACTACTTCTTCTTTGTTGGTTTGGACTATGGTTTTGTTGGGTAGATACCCAGAATGGCAAGCTAGAGCTAGAGAAGAAGTTTTGCAAGTTTTCGGTAACCAAAACCCAAACAACGAAGGTTTGTCTCAATTGAAGATCGTTACTATGATCTTGTACGAAGTTTTGAGATTGTTCCCACCATTGATCTACTTCAACAGAGCTTTGAGAAAGGACTTGAAGTTGGGTAACTTGTTGTTGCCAGAAGGTACTCAAATCTCTTTGCCAATCTTGTTGATCCACCAAGACCACGACTTGTGGGGTGACGACGCTAAGGAATTCAAGCCAGAAAGATTCGCTGAAGGTATCGCTAAGGCTACTAAGGGTCAAGTTTCTTACTTCCCATTCGGTTGGGGTCCAAGAATCTGTTTGGGTCAAAACTTCGCTTTGTTGGAAGCTAAGATCGCTGTTTCTTTGTTGTTGCAAAACTTCTCTTTCGAATTGTCTCCAAACTACGTTCACGTTCCAACTACTGTTTTGACTTTGCAACCAAAGAACGGTGCTTCTATCATCTTGCACAAGTTGTAA |
| *βAS (Zhu et al., 2017)* | ATGTGGAGATTGAAGATCGCTGAAGGTGGTAAGGACCCATACATCTACTCTACTAACAACTTCGTTGGTAGACAAACTTGGGAATACGACCCAGACGGTGGTACTCCAGAAGAAAGAGCTCAAGTTGACGCTGCTAGATTGCACTTCTACAACAACAGATTCCAAGTTAAGCCATGTGGTGACTTGTTGTGGAGATTCCAAATCTTGAGAGAAAACAACTTCAAGCAAACTATCGCTTCTGTTAAGATCGGTGACGGTGAAGAAATCACTTACGAAAAGGCTACTACTGCTGTTAGAAGAGCTGCTCACCACTTGTCTGCTTTGCAAACTTCTGACGGTCACTGGCCAGCTCAAATCGCTGGTCCATTGTTCTTCTTGCCACCATTGGTTTTCTGTATGTACATCACTGGTCACTTGGACTCTGTTTTCCCAGAAGAATACAGAAAGGAAATCTTGAGATACATCTACTACCACCAAAACGAAGACGGTGGTTGGGGTTTGCACATCGAAGGTCACTCTACTATGTTCTGTACTGCTTTGAACTACATCTGTATGAGAATCTTGGGTGAAGGTCCAGACGGTGGTCAAGACAACGCTTGTGCTAGAGCTAGAAAGTGGATCCACGACCACGGTGGTGTTACTCACATCCCATCTTGGGGTAAGACTTGGTTGTCTATCTTGGGTGTTTTCGACTGGTGTGGTTCTAACCCAATGCCACCAGAATTCTGGATCTTGCCATCTTTCTTGCCAATGCACCCAGCTAAGATGTGGTGTTACTGTAGATTGGTTTACATGCCAATGTCTTACTTGTACGGTAAGAGATTCGTTGGTCCAATCACTCCATTGATCTTGCAATTGAGAGAAGAATTGTTCACTGAACCATACGAAAAGGTTAACTGGAAGAAGGCTAGACACCAATGTGCTAAGGAAGACTTGTACTACCCACACCCATTGTTGCAAGACTTGATCTGGGACTCTTTGTACTTGTTCACTGAACCATTGTTGACTAGATGGCCATTCAACAAGTTGGTTAGAGAAAAGGCTTTGCAAGTTACTATGAAGCACATCCACTACGAAGACGAAACTTCTAGATACATCACTATCGGTTGTGTTGAAAAGGTTTTGTGTATGTTGGCTTGTTGGGTTGAAGACCCAAACGGTGACGCTTTCAAGAAGCACTTGGCTAGAGTTCCAGACTACTTGTGGGTTTCTGAAGACGGTATGACTATGCAATCTTTCGGTTCTCAAGAATGGGACGCTGGTTTCGCTGTTCAAGCTTTGTTGGCTACTAACTTGGTTGAAGAAATCGCTCCAACTTTGGCTAAGGGTCACGACTTCATCAAGAAGTCTCAAGTTAGAGACAACCCATCTGGTGACTTCAAGTCTATGTACAGACACATCTCTAAGGGTTCTTGGACTTTCTCTGACCAAGACCACGGTTGGCAAGTTTCTGACTGTACTGCTGAAGGTTTGAAGTGTTGTTTGTTGTTGTCTATGTTGCCACCAGAAATCGTTGGTGAAAAGATGGAACCAGAAAGATTGTACGACTCTGTTAACGTTTTGTTGTCTTTGCAATCTAAGAAGGGTGGTTTGTCTGCTTGGGAACCAGCTGGTGCTCAAGAATGGTTGGAATTGTTGAACCCAACTGAATTCTTCGCTGACATCGTTGTTGAACACGAATACGTTGAATGTACTGGTTCTGCTATCCAAGCTTTGGTTTTGTTCAAGAAGTTGTACCCAGGTCACAGAAAGAAGGAAATCGAAAACTTCATCGCTAACGCTGTTAGATTCTTGGAAGACACTCAAACTGCTGACGGTTCTTGGTACGGTAACTGGGGTGTTTGTTTCACTTACGGTTCTTGGTTCGCTTTGGGTGGTTTGGCTGCTGCTGGTAAGACTTTCGCTAACTGTGCTGCTATCAGAAAGGCTGTTAAGTTCTTGTTGACTACTCAAAGAGAAGACGGTGGTTGGGGTGAATCTTACTTGTCTTCTCCAAAGAAGATCTACGTTCCATTGGAAGGTTCTAGATCTAACGTTGTTCACACTGCTTGGGCTTTGATGGGTTTGATCCACGCTGGTCAAGCTGAAAGAGACCCAGCTCCATTGCACAGAGCTGCTAAGTTGATCATCAACTCTCAATTGGAAGAAGGTGACTGGCCACAACAAGAAATCACTGGTGTTTTCATGAAGAACTGTATGTTGCACTACCCAATGTACAGAGACATCTACCCAATGTGGGCTTTGGCTGAATACAGAAGAAGAGTTCCATTGCCATCTACTCCAGTTTGTTTGACTTAA |
| Gu*CPR1(Zhu et al., 2017)* | ATGACTTCGAATTCCGATTTGGTTCGCACCATTGAGTCGGTGCTGGGCGTTTCACTCGGCGATTCGGTCTCCGATTCGCTCGTTCTGATAGCGACTACCTCCGTCGCCGTCATAATTGGGCTCCTTGTGTTCCTGTGGAAGAAATCTTCGGATCGGAGCAGGGAGGTGAGGCCGGTGATTGTGCCGAAGTCGTTGGTGAAGGATGAAGACGATGATGTCGACGTTGCCTCCGGGAAGACTAAGGTTACTGTTTTCTTCGGTACTCAGACTGGTACTGCTGAGGGCTTCGCTAAGGCATTGGCGGACGAGATCAAGGCAAGATATGAAAAAGCATATGTCAAAGTTGTTGATTTGGATGACTATGCAATGGATGATGATCAATATGAGGAGAAGCTGAAGAAAGAAACTCTTGCATTTTTCATGCTGGCAACTTATGGAGATGGAGAGCCAACTGACAATGCTGCAAGATTCTACAAATGGTTTACTGAGGGTAAAGAGGAGAGGGGCACCTGGCTTCAACAGCTCACATATGGAGTTTTTGGCCTAGGTAACAAGCAATATGAACATTTTAATAAGATAGGTAAAGTTGTTGATGAAGACCTTAGTGAACAAGGTGCAAAGCGTCTTGTTCAACTTGGACTAGGTGATGATGATCAATCCATTGAGGATGATTTTTCTGCCTGGAAAGAATCTCTGTGGCCTGAGTTGGATCAGTTGCTCCGAGATGAGGATGATGTGAATACTGTTTCTACTCCCTATACTGCTGCTATTCCTGAATATCGAGTAGTGATTCATGACTCCACTGTCACACCATCCTATGATAATCAATTCAGCGCAGCAAATGGGGGTGCTGTATTTGATATTCATCATCCTTGCAGGGTAAATGTTGCTGTTAAAAGGGAGCTTCACAAACCTCAGTCTGACCGTTCCTGCATACATTTGGAGTTTGATATATCGGGGACTGGCATAACATATGAAACTGGAGACCATGTGGGTGTTTATGCTGAGAACTGTGATGAAACTGTTGAAGAAGCTGGGAAGTTGTTGGGTCAAAATTTAGATTTGCTGTTTTCTCTTCACACTGATAACGAGGATGGCACTTCCCTTGGAGGCTCTCTGCTACCTCCTTTCCCTGGTCCTTGCACACTGCGTACGGCGTTAGCACGTTATGCAGATCTCTTGAACCCTCCACGAAAGGCTGCGTTAGTTGTATTAGCTGCTCATGCTTCTGAACCTAGTGAGGCAGAAAGATTGAAGTTCCTCTCCTCTCCTCAGGGGAAGGATGAGTACTCCAAATGGGTGGTTGGAAGCCAGAGAAGTCTCCTTGAGGTGATGGCTGAGTTTCCATCAGCAAAACCTCCACTTGGTGTGTTTTTCGCTGCCATAGCCCCTCGTTTACAGCCTCGTTATTATTCTATTTCATCCTCTCCTAGGTTTGCCCCACAAAGGGTACATGTAACTTGTGCCCTGGTGTATGGTCCAACTCCCACTGGCAGAATTCACAAAGGAGTATGCTCAACCTGGATGAAGAATGCTATTCCCTTAGAGGAAAGCCGTGACTGTGGCTGGGCTCCCATTTTTATCAGGCCATCAAATTTCAAGCTACCAGCCGATCATTCGATTCCTATTATTATGGTTGGACCTGGTACTGGTTTGGCACCTTTTAGGGGATTTTTACAGGAAAGATTTGCCCTCAAAGAGGATGGTGTTCAACTTGGTCCTTCATTACTCTTCTTTGGATGCAGGAACCGTCAAATGGATTTTATTTATGAGGATGAGCTAAAGAATTTTGTGGAACAAGGTTCTCTGTCAGAGTTGATAGTTGCATTCTCTAGAGAGGGGCCTGAAAAGGAGTATGTTCAACACAAGATGATGGATAAAGCAGCATACCTGTGGAGTCTGATTTCTCAGGGAGGTTATCTTTATGTCTGTGGTGATGCCAAGGGTATGGCCAGAGATGTTCATCGAATTCTTCATACCATTGTCCAGCAGCAGGAAAATGTGGAGTCGTCAAAGGCGGAGGCTATAGTGAAAAAACTCCAGATGGATGGACGTTACCTCAGGGATGTCTGGTGA |
| Gu*UGT73F15*  *(Codon optimized for S. cerevisiae)* | ATGGGGACCCACAGTAAAGATACTTGCAGGAATTGGCCAAAGAGTAGAACAAATAACTGTAGGGAAAAGCCTAAATTTTTGGCCCCGATGGATCTGGAACGGGAAGAGATAGAGAAGCCACTTAAACTCTACTTCATCCATTACCTAGCAGCTGGTCACATGATCCCTCTCTGCGACATAGCCACTCTCTTCGCCTCACGTGGCCACCACGTGACCATCATCACCACTCCCTCCAACGCCCAAACCCTCCGCAGATCCATCCCTTTCAACGACTACCACCACCTCTGCCTCCACACCGTCCCATTCCCTTCCCAAGAGGTTGGCCTCCCCGACGGCGTGGAAAGCCTCTCCTCCGTAAGCGACCTTGACAACCTCGCCAAGGTGTTCCAAGCCACCACTCTGCTCCGAACACCCATCGAACACTTCGTGGAGGAGAACCCACCGGACTGCATCGTCGCCGACTTTATATACCAATGGGTTGATGAACTAGCGAACAAGCTCAACATCCCCAGGCTCGCCTTCAATGGTTTCTCACTCTTCGCAATCTGCGCCATAGAATCTGTTAAGGCCCACTCACTCTATGCCTCTGGTTCTTTCGTCATTCCGGGTCTTCCACACCCCATCGCCATGAACGCAGCGCCGCCGAAACAGATGAGCGACTTCCTCGAGTCTATGCTGGAGACAGAGCTCAAAAGCCATGGCCTCATCGTCAACAACTTCGCCGAACTCGACGGAGAAGAGTACATCGAACACTACGAGAAAACCACGGGGCACAAAGCTTGGCATCTTGGCCCTGTTTCTCTGATTCGCAGAACCGCACAGGAGAAAGCAGAGAGGGGAGAAAAGAGCGCGGTGAGTGTGCACGAGTGCCTGAGTTGGCTCGACTCGAAGCGAGACGACTCAGTGCTTTACATATGCTTTGGGAGCCTCTGTCATTTCTCGGATAAACAGCTTTATGAGATAGCGTGTGGGGTAGAAGCTTCGGGTCACGAATTCATATGGGTTGTTCCTGAGAAGAAGGGGAAGGAGGATGAGAGTGAAGAAGAGAAGGAAAAATGGATGCCAAAGGGATTCGAAGAGAGGAAGAAGGGGTTGATAATTAGGGGGTGGGCCCCACAGGTTCTAATCCTGAGCCACCGTGCTGTGGGTGCGTTCGTGACGCATTGCGGGTGGAACTCCACCGTGGAGGCTGTTTCGGCGGGGGTTCCGATGATCACGTGGCCGGTGCATGGGGAGCAATTCTACAACGAGAAGCTGGTAACTCGGGTGCGGGGGATCGGGGTGGAGGTGGGTGCGGAAGAGTGGAGCGCCATTGGTTTTGGGGAGAGGGAGAAGGTTGTGTGCAGAGAGAGCATAGAGAAGGCGGTGAGGAGGTTGATGGACGGTGGTGATGAGGCCGAGAAAATCAGACGGTGTGCACGAGAGTTTGGGGATAAGGCTATGCAGGCTGTTCAGGAAGGTGGTTCCTCACACAATAATTTAACGGCCCTGATTGATGATCTAAGAAGATTGAGGGATCGCAAGGTTCTAGATTAA |
| Cr*CYB5(Qu et al., 2015)* | ATGGCGTCGGATCAGAAATTGCACAAATTTGATGAAGTATCGAAGCATAACAAAACAAAGGATTGCTGGCTGATCATCAACGGAAAGGTTTATGATGTGACCCCCTTTATGGATGATCATCCTGGAGGTGATGAAGTCTTGCTTTCAGCCACTGGAAAAGATGCTACAAATGATTTCGAAGATGTGGGTCACAGTGATTCTGCCAGAGAGATGATGGATAAATACTACATTGGGGAGATGGACATGGCAACAGTTCCCCTTAAACGCACCTACATTCCACCCCAACAAGCTCAATACAATCCTGATAAGACTCCGGAATTCGTAATCAAGATTCTACAGTTCCTTGTACCCCTCTTGATCTTGGGTTTGGCCTTTGCTGTCCGACACTATACTAAGGAGAAGTAA |
| mut*CYP72A63 W205A T338S* | ATGGAAGTTTTCATGTTCCCAACTGGTACTACTGTTATCATCTCTGTTTTGTCTGTTTTGTTGGCTGTTATCCCATGGTACTTGTTGAACAAGTTGTGGTTGAAGCCAAAGAGATTCGAAAAGTTGTTGAAGGCTCAAGGTTTCCAAGGTGAACCATACAACTTGTCTGTTTTGAAGGACAAGTCTAAGCAAAACTACATGTTGAAGTTGCAACAAGAAGACAAGTCTAAGTCTATCGGTTTGTCTAAGGAAGCTGCTCCATCTATCTTCACTCCAGTTCACCAAACTGTTAGAAAGTACGGTAACAACTCTTTCTTGTGGGAAGGTACTACTCCAAGAGTTATCATCACTGACCCAGACCAAATCAAGGACGTTTTCAACAAGATCGACGACTTCCCAAAGCCAAAGTTGAGATCTATCGCTAAGTACTTGTCTGTTGGTATCTTGGACCACGAAGGTAAGAAGTGGGCTAAGCACAGAAAGATCGCTAACCCAGCTTTCCACTTGGAAAAGTTGAAGGTTATGTTGCCAGCTTTCTCTCACTCTTGTAACGAAATGATCTCTAAGTGGAAGGAATTGTTGTCTTCTGACGGTACTTGTGAAATCGACGTTGCTCCATCTTTGCAAAACTTCACTTGTGACGTTATCTCTAGAACTGCTTTCGGTTCTTCTTACGCTGAAGGTACTAAGTTGTTCCAATTGTTGAAGAAGCAAGGTTTCTTGTTGATGACTGGTAGACACACTAACAACCCATTGTGGGGTTTGTTGGCTACTACTACTAAGACTAAGATGAAGGAAATCGACAGAGAAATCCACGACTCTTTGGAAGGTATCATCGAAAAGAGAGAAAAGGCTTTGAAGAACGGTGAAACTACTAACGACGACTTGTTGGGTATCTTGTTGCAATCTAACCACGCTGAAAAGCAAGGTCAAGGTAACTCTAAGAACATCGGTATGACTACTCAAGACGTTATCGACGAATGTAAGTTGTTCTACTTGGCTGGTCAAGAAAGTACTTCTTCTTTGTTGGTTTGGACTATGGTTTTGTTGGGTAGATACCCAGAATGGCAAGCTAGAGCTAGAGAAGAAGTTTTGCAAGTTTTCGGTAACCAAAACCCAAACAACGAAGGTTTGTCTCAATTGAAGATCGTTACTATGATCTTGTACGAAGTTTTGAGATTGTTCCCACCATTGATCTACTTCAACAGAGCTTTGAGAAAGGACTTGAAGTTGGGTAACTTGTTGTTGCCAGAAGGTACTCAAATCTCTTTGCCAATCTTGTTGATCCACCAAGACCACGACTTGTGGGGTGACGACGCTAAGGAATTCAAGCCAGAAAGATTCGCTGAAGGTATCGCTAAGGCTACTAAGGGTCAAGTTTCTTACTTCCCATTCGGTTGGGGTCCAAGAATCTGTTTGGGTCAAAACTTCGCTTTGTTGGAAGCTAAGATCGCTGTTTCTTTGTTGTTGCAAAACTTCTCTTTCGAATTGTCTCCAAACTACGTTCACGTTCCAACTACTGTTTTGACTTTGCAACCAAAGAACGGTGCTTCTATCATCTTGCACAAGTTGTAA |
| At*UDH(Oka and Jigami, 2006)* | ATGGTGAAGATATGCTGCATAGGAGCTGGTTATGTGGGTGGTCCAACCATGGCGGTGATGGCTCTTAAGTGTCCTGAGATTGAAGTAGTCGTTGTGGATATCTCTGAACCAAGGATCAATGCTTGGAACAGTGATAGGCTTCCTATTTACGAGCCGGGATTGGAAGATGTGGTGAAACAATGCAGAGGGAAAAACCTCTTCTTTAGCACAGACGTGGAGAAACATGTATTTGAGAGTGATATTGTATTTGTCTCAGTTAACACTCCAACCAAAACACAAGGTCTTGGTGCTGGCAAAGCTGCTGATCTTACTTACTGGGAGAGTGCTGCTCGGATGATCGCTGATGTCTCCAAATCTAGCAAAATCGTTGTTGAGAAATCCACGGTTCCTGTGAGGACAGCAGAGGCTATTGAAAAGATACTGACACATAACAGCAAAGGCATAGAGTTTCAGATTCTCTCTAACCCTGAATTTCTTGCTGAGGGTACTGCAATTAAGGATCTTTATAACCCAGACCGTGTGTTGATTGGTGGTAGGGATACTGCAGCAGGGCAAAAGGCTATTAAAGCTTTAAGAGATGTTTATGCTCATTGGGTTCCAGTGGAACAAATCATTTGCACGAACCTGTGGTCCGCTGAGCTCTCTAAGCTTGCAGCAAATGCATTCTTAGCTCAGAGGATATCATCTGTCAATGCCATGTCAGCTCTATGTGAGGCAACTGGCGCTGATGTTACACAAGTTGCGCATGCCGTGGGTACAGATACTAGAATTGGTCCAAAGTTCTTGAATGCTAGTGTTGGTTTTGGTGGATCATGTTTCCAAAAGGACATCCTAAATCTTATCTATATTTGTGAATGCAACGGCTTGCCCGAAGCAGCTAATTACTGGAAACAAGTCGTAAAGGTGAACGACTATCAGAAAATACGGTTTGCAAACCGGGTTGTTTCTTCAATGTTTAACACAGTCTCGGGCAAGAAAATCGCGATCCTCGGTTTTGCCTTCAAGAAGGACACAGGTGACACGAGAGAGACTCCAGCGATTGATGTTTGTAACAGATTAGTTGCAGACAAGGCCAAGCTGAGCATATACGACCCACAAGTTCTTGAAGAACAGATCAGAAGAGATCTTTCCATGGCTAGGTTTGACTGGGACCACCCTGTTCCTCTTCAGCAGATTAAAGCTGAAGGTATCTCAGAGCAAGTGAATGTCGTCTCAGATGCTTACGAGGCAACTAAAGATGCGCACGGCCTATGTGTCTTAACCGAATGGGATGAGTTTAAATCCTTGGACTTCAAGAAAATCTTTGACAATATGCAGAAACCAGCTTTTGTGTTCGATGGTAGGAATGTTGTTGATGCAGTGAAGCTGCGTGAGATCGGTTTCATCGTCTACTCCATTGGTAAACCGCTTGATTCATGGCTCAAGGATATGCCTGCTGTGGCATGA |

**Supplementary Table S2** List of plasmids used in this study

| Plasmid | Description | Source |
| --- | --- | --- |
| pRS41K-SpCas9 | CEN/ARS; G418; *AmpR*; P*_TEF1_-SpCas9-*T*_TEF1_;* | (Lian et al., 2017) |
| p423-SpSgH | 2μ; *HIS3; AmpR;* P*_SNR52_-*T*_SUP4_* | (Lian et al., 2017) |
| p426-SpSgH | 2μ; *URA3; AmpR;* P*_SNR52_-*T*_SUP4_* | (Lian et al., 2017) |
| pESC-URA | 2μ; *URA3; AmpR;* P*_GAL1_-*MCS1*-T_CYC1_;* P*_GAL10_-*MCS2*-T_ADH1_* (NCBI Accession AF063585) | Agilent Technologies, Inc |
| pESC-LEU | 2μ; *LEU2; AmpR;* P*_GAL1_*-MCS1-*T_CYC1_;* P*_GAL10_*-MCS2-*T_ADH1_*  (NCBI Accession AF063849) | Agilent Technologies, Inc |
| pESC-TRP | 2μ; *TRP1; AmpR;* P*_GAL1_*-MCS1-*T_CYC1_;* P*_GAL10_*-MCS2-*T_ADH1_*  (NCBI Accession AF063848) | Agilent Technologies, Inc |
| pESC-HIS | 2μ; *HIS3; AmpR;* P*_GAL1_*-MCS1-*T_CYC1_;* P*_GAL10_*-MCS2-*T_ADH1_*  (NCBI Accession AF063850) | Agilent Technologies, Inc |
| pUMI-22 | 2μ; *HIS3; AmpR;* P*_GAL2_*-MCS1-*T_CYC1_;* P*_GAL7_*-MCS2-*T_ADH1_* | (Lv et al., 2016) |
| pESC-LEU-Gu*UGT73F15-AtUDH* | 2μ; *LEU2; AmpR;* P*_GAL1_*-*GuUGT73F15*-*T_CYC1_;* P*_GAL10_*-At*UDH*-*T_ADH1_* | This study |
| pESC-URA-Cu*CPR1*-Cr*CYB5* | 2μ; *URA3; AmpR;* P*_GAL1_-GuCPR1*-*T_CYC1_;* P*_GAL10_*-Cr*CYB5*-*T_ADH1_* | This study |
| pESC-HIS-*ERG9*-*ERG20* | 2μ; *HIS3; AmpR;* P*_GAL1_-ERG9*-*T_CYC1_;* P*_GAL10_*-*ERG20-T_ADH1_* | This study |
| pESC-TRP-*CYP88D6-CYP72A63* | 2μ; *TRP1; AmpR;* P*_GAL1_-CYP88D6*-*T_CYC1_;* P*_GAL10_*-*CYP72A63-T_ADH1_* | This study |
| pESC-TRP-*UNI25647-CYP72A63* | 2μ; *TRP1; AmpR;* P*_GAL1_-CYP88D6*-*T_CYC1_;* P*_GAL10_*-*CYP72A63-T_ADH1_* | This study |
| pESC-TRP-*UNI25647-mtCYP72A63* | 2μ; *TRP1; AmpR;* P*_GAL1_-UNI25647*-*T_CYC1_;* P*_GAL10_*-mut*CYP72A63(W205A/T338S)-T_ADH1_* | This study |
| pESC-TRP-*UNI25647* | 2μ; *TRP1; AmpR;* P*_GAL1_-UNI25647-T_CYC1_;* P*_GAL10_*-*CYP72A63-T_ADH1_* | This study |
| pUMI-β*AS* | 2μ; *URA3; AmpR;* P*_GAL7_*-*βAS*- *T_TPS1_;* P*_GAL2_*-*T_TPK1_* | This study |
| pESC-URA-*ERG1-tHMG1* | 2μ; *URA3; AmpR;* P*_GAL1_* -*ERG1*-*T_CYC1_;* P*_GAL10_* -*tHMG1*-*T_ADH1_* | This study |
| pESC-HIS-*UGP1*-At*UDH* | 2μ; *HIS3; AmpR;* P*_GAL1_* -*UGP1*-*T_CYC1_;* P*_GAL10_* -At*UDH*-*T_ADH1_* | This study |
| pESC-HIS-*PGM1*-*PGM2* | 2μ; *HIS3; AmpR;* P*_GAL1_* -*PGM1*-*T_CYC1_;* P*_GAL10_* -*PGM2*-*T_ADH1_* | This study |
| pIYC04-*ZWF1*-*GAPN* | 2μ; *HIS3; AmpR;* P*_PGK1_* -*ZWF1*-*T_CYC1_;* P*_TEF1_*-*GAPN*-*T_ADH1_* | This study |

# Supplementary Table S3 List of primers used in this study

| **Primer name** | **Sequence (5′-3′)** |
| --- | --- |
| **For plasmids construction** |  |
| *gal7-βAS-F* | CCGTAATACGACTCACTATAGGGCCCGGGATGTGGAGATTGAAGATCGCTG |
| *tps1t-βAS-R* | AGCTAGCCGCGGTACCAAGCTTACTCGAGTTAAGTCAAACAAACTGGAGTAG |
| *adh1t-ERG20-R* | CGTCATCCTTGTAATCCATCGATACTAGTCTATTTGCTTCTCTTGTAAACT |
| *gal10-ERG20-F* | TCGAATTCAACCCTCACTAAAGGATGGCTTCAGAAAAAGAAATTAG |
| *gal1-ERG9-F* | TAACGTCAAGGAGAAAAAACCCCGGATCCATGGGAAAGCTATTACAATTG |
| *cyc1t-ERG9-R* | AGCTAGCCGCGGTACCAAGCTTACTCGAGTCACGCTCTGTGTAAAGTG |
| *adh1t-CYP72A63-R* | CCTTGTAATCCATCGATACTAGTTTACAATTTATGCAAAATGATAGATG |
| *gal10-CYP72A63-F* | TCGAATTCAACCCTCACTAAAGGATGGAAGTTTTTATGTTTCCAA |
| *ga11-CYP88D6-F* | TAACGTCAAGGAGAAAAAACCCCGGATCCATGGAAGTACATTGGGTTTG |
| *cyc1-CYP88D6-R* | AGCTAGCCGCGGTACCAAGCTTACTCGAGCTAAGCACATGAGACCTTC |
| *ga11-UNI25647-F* | TAACGTCAAGGAGAAAAAACCCCGGATCCATGGAAGTACATTGGGTTTG |
| *cyc1-UNI25647-R* | AGCTAGCCGCGGTACCAAGCTTACTCGAGCTAAGCACATGAGACCTTC |
| *adh1t-tHMG1-R* | TCCATCGATACTAGTGCGGCCGCTTAGGATTTAATGCAGGTGAC |
| *gal10-tHMG1-F* | ATTTTTGAAAATTCGAATTCATGGCTGCAGACCAATTGGTG |
| *gal1-ERG1-F* | TAACGTCAAGGAGAAAAAACCCCGGATCCATGGCTCCGACGATATTCG |
| *cyc1t-ERG1-R* | AGCTAGCCGCGGTACCAAGCTTACTCGAGTCATTGAGGAGAAGAAGAAGAAG |
| *gal1-*Gu*CPR1*-F | TAACGTCAAGGAGAAAAAACCCCGGATCCATGACTTCGAATTCCGATTTG |
| *cyc1-*Gu*CPR1*-R | GCTAGCCGCGGTACCAAGCTTACTCGAGTCACCAGACATCCCTGAGGTAA |
| *gal10-*Cr*CYB5*-F | TTGTAATCCATCGATACTAGTTTACTTCTCCTTAGTATAGTG |
| *tadh1-*Cr*CYB5*-R | CGAATTCAACCCTCACTAAAGGATGGCGTCGGATCAGAAATT |
| *tadh1-*At*UDH-R* | CCTTGTAATCCATCGATACTAGTTCATGCCACAGCAGGCATATC |
| *gal10-*At*UDH-F* | TCGAATTCAACCCTCACTAAAGGATGGTGAAGATATGCTGCATAG |
| *gal1-*Gu*UGT73F15-F* | TAACGTCAAGGAGAAAAAACCCCGGATCCATGGGGACCCACAGTAAAG |
| *cyc1-*Gu*UGT73F15-R* | AGCTAGCCGCGGTACCAAGCTTACTCGAGTTAATCTAGAACCTTGCGATC |
| *gal1-PGM1-F* | TAACGTCAAGGAGAAAAAACCCCGGATCCATGTCACTTCTAATAGATTC |
| *cyc1-PGM1-R* | AGCTAGCCGCGGTACCAAGCTTACTCGAGCTATGTGCGGACTGTTGG |
| *gal10-PGM2-F* | TCGAATTCAACCCTCACTAAAGGGCGGCCGCATGTCATTTCAAATTGAAACG |
| *adh1-PGM2-R* | CGTCATCCTTGTAATCCATCGATACTAGTTTAAGTACGAACCGTTGGTTC |
| *gal1-UGP1-F* | TAACGTCAAGGAGAAAAAACCCCGGATCCATGTCCACTAAGAAGCACAC |
| *cyc1-UGP1-R* | AGCTAGCCGCGGTACCAAGCTTACTCGAGTCAATGTTCCAAGATTTGCA |
| *gal1-ZWF1*-F | AACCCCGGATCCATGAGTGAAGGCCCCGTCAAA |
| *cyc1-ZWF1*-R | GCTTACTCGAGCTAATTATCCTTCGTATCTT |
| *gal10-GAPN*-F | GCTCAGATCAGATCTTGTTTTACTAGTGTCACTTTATGTCAAAGACAA |
| *adh1-GAPN*-R | TCTAATCTAAGTTTTAATTACAAGCGGCCGCATGACAAAACAATACAAAAACT |
| **For genome integration** |  |
| INT1-*CYP88D6*-*CYP72A63*-F | CTATGTGACGCTGTGTATTCTTTGTTGTAGTTATGCTCCATTCAGGCTGCGCAACTGTT |
| INT1-*CYP88D6*-*CYP72A63*-R | TGTACGCTATACATTTACGTGCTGAGCTCCTAGGAAAGCTAGTGAGCTGATACCGCTCG |
| INT2-*GuUGT73F15-AtUDH*-F | GTTTTCTTATTTCTTTCTTTTTAAAAAACTTTCTTAATATTTCAGGCTGCGCAACTGTT |
| INT2-*GuUGT73F15-AtUDH*-R | TTGAGAAAAAAAGTGTATATCATTACATTACTTTACACCAAGTGAGCTGATACCGCTCG |
| INT3-*GuCPR*-*CrCYB5*-F | TATGTATTTTGCAGCTTCTACTTTCTGCAATGTGATGCCGTTCAGGCTGCGCAACTGTT |
| INT3-*GuCPR*-*CrCYB5*-R | CAGTAACTAATCGCAAACAAATCAGGCATCTGTGTATATTAGTGAGCTGATACCGCTCG |
| INT4-*ERG1*-*tHMG1*-F | TTACGTGTCATTTATTATGGGTTCAGAAATTATGTGTTAATTCAGGCTGCGCAACTGTT |
| INT4-*ERG1*-*tHMG1*-R | ACAATTTTGGTGGCGTTGAAATTGATGCCGGAATTTGCCCAGTGAGCTGATACCGCTCG |
| INT5-*bAS*-F | TTACGTGTCATTTATTATGGGTTCAGAAATTATGTGTTAAAGGTGATATCAGATCCACG |
| INT5-*bAS*-R | ACAATTTTGGTGGCGTTGAAATTGATGCCGGAATTTGCCCACGAGGCAAGCTACTAGAC |
| INT6-*ERG9*-*ERG20*-F | TCTAGTATCAAAGAAACTTACTATGACGCAGTTTAGGATCGAGCGACCTCATGCTATAC |
| INT6-*ERG9*-*ERG20*-R | GACACTGAATAAACAAGGGGCTTTACGATGGAGTAGTAGACTTCGAGCGTCCCAAAACC |
| INT7-UNI*25647*-F | GAGAGAAATTAAACTTGGTTGGGGTTAATTATTTGATGGGTTCAGGCTGCGCAACTGTT |
| INT7-*UNI25647*-R | TATTGATAAAGGTTTTGTAGAATATTTATTATCGATACCTAGTGAGCTGATACCGCTCG |
| INT8-*UNI25647-CYP72A63(Mt)*-F | AGCAATAAACGTGAAATCTGTTTTGTATATATATTTGCAGGAGCGACCTCATGCTATAC |
| INT8-*UNI25647-CYP72A63(Mt)*-R | GGTTTTGGGACGCTCGAAGCGGATAATTTGAGCAATGATAGTATGAAATGCCAGATTAC |
| INT9-*UGD1-AtUDH1*-F | CTACCAAGGTTGTTGAGGGAACACTGGGGCAATAGGCTGTGAGCGACCTCATGCTATAC |
| INT9-*UGD1-AtUDH1*--R | CTGTTACTTCTTGCAGACATCAGACATACTATTGTAATTCCTTCGAGCGTCCCAAAACC |
| INT10-*PGM1-PGM2*-F | ACCGGTACCGGAGGAGACCGCTATAACCGGTTTGAATTTAGAGCGACCTCATGCTATAC |
| INT10-*PGM1-PGM2*--R | TTCGAATGATGAACTTGCTTGCTGTCAAACTTCTGAGTTGCTTCGAGCGTCCCAAAACC |
| ΔINT1-F | ACACATACTCCTGAATGGGTGCTTTCAAATCCCCTCTCCCTTCAGGCTGCGCAACTGTT |
| ΔINT1-R | TGTACGCTATACATTTACGTGCTGAGCTCCTAGGAAAGCTAGTGAGCTGATACCGCTCG |
| Δ*OPI1*:*ZWF1*-*GAPN*-F | ATTCAAAGGTAAAGAGGGTCCGATAATAATGGTAGTTCAATTCAGGCTGCGCAACTGTT |
| Δ*OPI1*:*ZWF1*-*GAPN*-R | AGCGATCTGCACTTAGCCAAGAAAGCATATCAGGCCAGAAAGTGAGCTGATACCGCTCG |
| Δ*EGH1-F* | TGCGATAAAGATGGCAACGAGATCCAATTGCGTGGTGTCAAGGTGATATCAGATCCACG |
| Δ*EGH1-R* | AGGGACTATCCAAATCTAAATGATTATCTGGTTTTATGAAACGAGGCAAGCTACTAGAC |
| **For PCR Verification of genome integration** |  |
| Conf-INT1-F | TATTACAGGTGAATCGCGCGCTAA |
| Conf-INT1-R | CGCGGGCACAGAATGGATAACTGCA |
| Conf-INT2-F | GATAACTAGCCCTGCAAGCGAATTC |
| Conf-INT2-R | ACATACGCGATACGTTGTCGATAA |
| Conf-INT3-F | GATCTGGTGATGAGTTCTGCCAGAA |
| Conf-INT3-R | GCTGCCGACCAACAACCATTACTG |
| Conf-INT4-F | AATGGTGATTCCGGCAGCTTCATA |
| Conf-INT4-R | CAATTGTTGACGATGGTTCCTACCT |
| Conf-INT5-F | GCTTGATCATTCCGCAGAATAACC |
| Conf-INT5-R | CGGCAATGCATACTTACTAAGAGC |
| Conf-INT6-F | CTATTTATAAACGTCACTAACTAGAAATAC |
| Conf-INT6-R | GAGCATATGATCTTATTTTTCCAC |
| Conf-INT7-F | CGTCTCTGGTGTCGTGTATATAAG |
| Conf-INT7-R | ATTCCTTACAGGATGCACGGAGGTA |
| Conf-INT8-F | GATTGGTTTATGATAGAATGAGCTG |
| Conf-INT8-R | GGGTGCCTTTACCATAAAACC |
| Conf-INT9-F | CATGGCCAATTTGGCAAATTG |
| Conf-INT9-R | CGGTCAAAGGGGAAATCAATG |
| Conf-INT10-F | GCGTCCTACAGCGTGATGA |
| Conf-INT·10-R | CCTACATCAGGACAGTAGTACC |
| Conf-Δ*INT1*-F | CGGAAATGTATTCCCGACCCCCTTAC |
| Conf-Δ*INT1*-R | ATGCAACCGGGTAAAATCGGGCGTT |
| Conf-Δ*OPI1*-F | CGGAAATGTATTCCCGACCCCCTTAC |
| Conf-Δ*OPI1*-F | ATGCAACCGGGTAAAATCGGGCGTT |
| Conf-Δ*EGH1*-F | ATGCCTGCCAAAATACACATTTCTG |
| Conf-Δ*EGH1*-F | TGTAGGGACAGTTATCTGTTCAGGTG |

* Restriction sites are underlined.

**Supplementary Table S4** List of gRNAs used in this study

| **SgRNAs** | **Sequences (5′-3′)** | **Chromosomal loci** |
| --- | --- | --- |
| Sg-INT1 | CATGAGCAGCCACTGTATCG | ChrXIV: 298,004-298,023 |
| Sg-INT2 | ACACGTTTGTGGTTATAAGG | ChrIV: 561,137-561,156 |
| Sg-INT3 | TGGCATTGAGATTCCAACG | ChrXV: 544,591-544,610 |
| Sg-INT4 | GTAAAACACCTATAGCACTG | ChrVII: 320,896-320,915 |
| Sg-INT5 | GTTGAAATATAAGTAACCCT | ChrX: 415,611-415,630 |
| Sg-INT6 | CCTGTGGTGACTACGTATCC | ChrXIV: 262,867-262,886 |
| Sg-INT7 | TATATTAATTTGCAACCGCA | ChrXII: 498,543-498,562 |
| Sg-INT8 | GCGCCACAGTTTCAAGGGTC | ChrXI:V:28,250-28,269 |
| Sg-Δ*INT1* | GAAATAGGACTTGATCTTGG | ChrXIV:297922-297941 |
| Sg-Δ*OPI1* | TGTCGCGGGCGATTGCCAAG | ChrVIII: 67,100-67,119 |
| Sg-Δ*EGH1* | AGGATTAACAGCGTACAACA | ChrIX:371,049-371,068 |

#

# Supplementary Table S5 List of strains used in this study.

| **Name** | **Description** | **Source** |
| --- | --- | --- |
| BY4741-C-04  Trp his marker | BY4741, Δ*LPP1*:T*_CYC1_*-*ERG10­*-P*_GAL1_*-P*_GAL10_*-*HMGS*-T*_ADH1_*;  Δ*HO*::T*_TPS1_*-*tHMG1­*-P*_GAL7_*-P*_GAL2_*-*ERG12*-T*_PGK1_*;  Δ*DPP1*::T*_CYC1_*-*tHMG1­*-P*_GAL1_*-P*_GAL10_*-*PMK*-T*_ADH1_*;  Δ*GAL80*::T*_TPS1_*-*MVD1­*-P*_GAL7_*-P*_GAL2_*-*IDI1*-T*_PGK1_* | (Lv et al., 2016) |
| CI04-1-1 | BY4741-C-04, INT1::T*_CYC1_*-*CYP88D6­*-P*_GAL1_*-P*_GAL10_*-*CYP72A63*-T*_ADH1_*; INT2::T*_CYC1_*-Gu*UGT73F15­*-P*_GAL1_*-P*_GAL10_*-At*UDH*-T*_ADH1_* | This study |
| CI04-3 | CI04-1-1, INT3::T*_CYC1_*-Gu*CPR1­*-P*_GAL1_*-P*_GAL10_*-Cr*CYB5*-T*_ADH1_*;  INT4::T*_CYC1_*-*tHMG1*-P*_GAL1_*-P*_GAL10_*-At*ERG1*-T*_ADH1_* | This study |
| CI04-4 | CI04-3, INT5::T*_TPS1_*-*βAS­*-P*_GAL7_* | This study |
| GA1 | CI04-4, INT6::T*_CYC1_*-*ERG9*-P*_GAL1_*-P*_GAL10_*-*ERG20*-T*_ADH1_* | This study |
| GA8 | GA1, ΔINT1; INT8::T*_CYC1_*-*UNI25647*-P*_GAL1_*-P*_GAL10_*-*CYP72A63*-T*_ADH1_* | This study |
| GA9 | GA1, ΔINT1; INT8::T*_CYC1_*-*UNI25647*-P*_GAL1_*-P*_GAL10_*-mut*CYP72A63*-T*_ADH1_* | This study |
| GA10 | GA8, INT7::T*_CYC1_*-*UNI25647*-P*_GAL1_* | This study |
| GA11 | GA9, INT7::T*_CYC1_*-*UNI25647*-P*_GAL1_* | This study |
| GA12 | GA10, Δ*OPI*::T*_CYC1_*-*ZWF1*-P*_PGK1_*-P*_TEF1_*-*GAPN*-T*_ADH1_* | This study |
| GA13 | GA11, Δ*OPI*::T*_CYC1_*-*ZWF1*-P*_PGK1_*-P*_TEF1_*-*GAPN*-T*_ADH1_* | This study |
| GA14 | GA13, INT9::T*_CYC1_*-At*UDH1*-P*_GAL1_*-P*_GAL10_*-*UGH1*-T*_ADH1_*;*_，_*  INT10::T*_CYC1_*-*PGM1*-P*_GAL1_*-P*_GAL10_*-*PGM2*-T*_ADH1_* | This study |
| GA15 | GA14, Δ*EGH1* | This study |

#



# Supplementary Figure S1. Verification of the glycosylation module (Module III). (A) HPLC profiles of the fermentation products of strain CI04-1-1 (Gu*UGT73F15* and At*UDH*) with (above) or without (bottom) the supplementation of GA. (B) MS spectra of GAMG (above) and GA (bottom) standards. Peak 1: GAMG; Peak 2: GA; Peak 3: GA-monoglucoside. (C) Mass spectra of GA-monoglucoside ([M-H]^-^=631.60) produced by CI041-1 feed by GA.

#
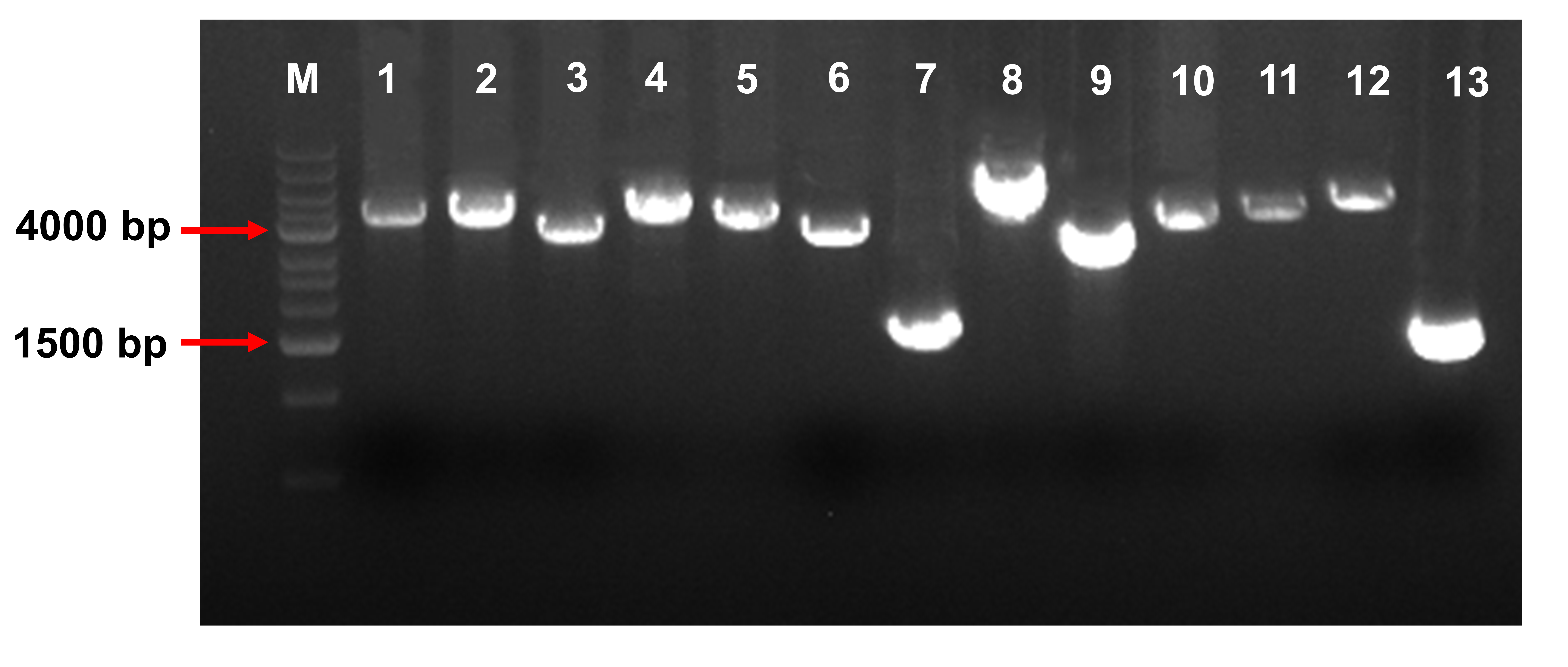
Supplementary Figure S2. PCR amplified gene cassettes for GA15. The 13 gene expression cassettes. M: marker;1: INT1-CYP88D6-CYP72A63, 2: INT2-UGT75F15-AtUDH; 3: INT3-GuCPR1-CYB5; 4: ERG1-tHMG1; 5: INT5-βAs; 6: INT6:ERG9-ERG20; 7: ΔINT1-pESC; 8: INT8-uni25467-mut72A63; 9: INT7-uni25467;10: Δopi: ZWF1-GAPN;11: INT9:UGD1-atUDH1;12: INT10:PGM1-PGM2;13: ΔEGH1.

**Reference**

Huang, Y., Li, D., Wang, J., Cai, Y., Dai, Z., Jiang, D., and Liu, C. (2019). GuUGT, a glycosyltransferase from Glycyrrhiza uralensis, exhibits glycyrrhetinic acid 3- and 30-O-glycosylation. *Royal Society open science* 6**,** 191121. doi:org/10.1098/rsos.191121

Lian, J., Hamedirad, M., Hu, S., and Zhao, H. (2017). Combinatorial metabolic engineering using an orthogonal tri-functional CRISPR system. *Nat Commun* 8**,** 1688. doi:org/10.1038/s41467-017-01695-x

Lv, X., Wang, F., Zhou, P., Ye, L., Xie, W., Xu, H., and Yu, H. (2016). Dual regulation of cytoplasmic and mitochondrial acetyl-CoA utilization for improved isoprene production in Saccharomyces cerevisiae. *Nat Commun* 7**,** 128-151.

Oka, T., and Jigami, Y. (2006). Reconstruction of de novo pathway for synthesis of UDP-glucuronic acid and UDP-xylose from intrinsic UDP-glucose in Saccharomyces cerevisiae. *The FEBS journal* 273**,** 2645-2657. doi:org/10.1111/j.1742-4658.2006.05281.x

Qu, Y., Easson, M.L., Froese, J., Simionescu, R., Hudlicky, T., and De Luca, V. (2015). Completion of the seven-step pathway from tabersonine to the anticancer drug precursor vindoline and its assembly in yeast. *Proceedings of the National Academy of Sciences of the United States of America* 112**,** 6224-6229. doi:org/10.1073/pnas.1501821112

Zhu, M., Wang, C., Sun, W., Zhou, A., Wang, Y., Zhang, G., Zhou, X., Huo, Y., and Li, C. (2017). Boosting 11-oxo-β-amyrin and glycyrrhetinic acid synthesis in Saccharomyces cerevisiae via pairing novel oxidation and reduction system from legume plants. *Metab Eng***,** 43-50.
